# Agroecological transition in Madrid’s extensive livestock systems: How expert-perceived NCPs guide transformation pathways

**DOI:** 10.1007/s13280-026-02361-7

**Published:** 2026-04-18

**Authors:** Beatriz Vizuete, Marina García-Llorente, Amanda Jiménez-Aceituno, Megan Meacham, Francisco M. Azcárate, Violeta Hevia, Inés Gutiérrez‐Briceño, Elisa Oteros‐Rozas

**Affiliations:** 1 Social-Ecological Systems Laboratory, Department of Ecology, Faculty of Science, Autonomous University of Madrid, Madrid, Spain; 2 Centro de Investigación en Biodiversidad y Cambio Global (CIBC-UAM), Universidad Autónoma de Madrid, Darwin 2, 28049 Madrid, Spain; 3 Stockholm Resilience Centre, Stockholm University, Stockholm, Sweden; 4 Social-Ecological Systems Institute, Faculty of Sustainability, Leuphana University Lüneburg, Lüneburg, Germany; 5 Terrestrial Ecology Group, Department of Ecology, Universidad Autónoma de Madrid, Darwin 2, 28049 Madrid, Spain; 6 Estación Biológica de Doñana (EBD), CSIC, Calle Américo Vespucio, 26, 41092 Seville, Spain

**Keywords:** Agroecology, Animal production, Ecosystem services, Expert analysis/criteria, Pastoral system, Sustainable transformation

## Abstract

In the Mediterranean region, the industrialization of livestock systems, driven by the replacement of extensive pasture-based systems with intensive input-dependent models, has triggered environmental degradation and socio-cultural changes. The transition of livestock systems toward agroecological practices can reduce external dependencies, enhance ecosystem contributions and strengthen socioecological resilience. This study analyzes experts’ perceptions of Nature’s Contributions to People (NCPs) provided by two extensive livestock models (conventional and agroecological) and nine agroecological practices in Madrid. Experts on extensive livestock farming with different profiles (*N* = 65) were surveyed to assess 14 NCPs. Cluster analysis identified four bundles of practices based on their functional similarities. Results showed that the agroecological model provides more NCPs within all domains, particularly regulating and non-material NCPs. We conclude that certain agroecological practices, such as active grazing management and production diversification, are pivotal in fostering systemic change by linking farm-scale interventions with broader agri-food transformations.

**Supplementary Information:**

The online version contains supplementary material available at 10.1007/s13280-026-02361-7.

## Introduction

Livestock systems have been affected in recent decades by the intensification and globalization of agri-food systems (Herrero et al. 2010). In many regions of the world, such as the Mediterranean basin, grazing was once the most ecologically sound practice for obtaining food from nature (Soto et al. 2016). However, increased productivity and specialization, together with the disconnection from local resource bases, have led to the replacement of extensive pasture-based livestock systems by increasingly input-intensive and indoor models (Naylor et al. 2005; Aubron et al. 2016; Bardgett et al. 2021). In the Mediterranean region, the intensification of livestock production systems is characterized by the decoupling of animal production from agriculture and silvopastoral systems, reduced livestock mobility (both spatially and temporally) and changes in the types of breeds raised. At an ecological level, these changes have led to increased dependence on external inputs, such as imported feed, which has contributed to large-scale deforestation for crop expansion and the decoupling of carbon and nitrogen cycles in space and time, making intensive systems largely unsustainable (Drinkwater and Snapp 2007; Ilea 2009). From a socio-cultural perspective, in many rural areas of Europe, livestock intensification has led to the abandonment of pastoral livestock farming (McDonald et al. 2000), increasing the vulnerability of peasant and pastoralist communities (Scoones 2021). At the same time, increased production and supply have led to decreased prices and therefore higher demand and consumption of meat and dairy products (Stoll-Kleeman and O’Riordan 2015; Soto et al. 2016). Moreover, it has accelerated the loss of traditional livestock and pasture management practices and knowledge, along with declines in biodiversity, genetic diversity of breeds (Azcárate and Peco 2012; Velado-Alonso et al. 2021), soil fertility, and ecosystem services such as fire prevention (Ruiz-Mirazo et al. 2012; Bernués et al 2016). Therefore, livestock production is considered one of the main drivers of global environmental change, highlighting the need to substantially transform animal production to reverse its negative impacts (Kleppel 2020).

In the European context, achieving sustainable agri-food systems for animal production is a multifactorial challenge that involves a series of systemic changes, including restoring extensive pasture-based livestock systems (Bernués et al. 2014; Capone et al. 2021) and adapting these systems to more sustainable management practices (Escribano et al. 2024). This transition can be guided by agroecological principles, such as reducing external inputs, improving social and animal health, preserving biodiversity, preserving territorial identities and promoting economic diversification (HLPE 2019; Wezel et al. 2020; Aguilera et al. 2020; Aguilera and Rivera-Ferre 2022). Pasture-based livestock systems have been associated with multiple benefits for societal well-being both at global (Leroy et al. 2024) and local levels (Peeters 2009; Plieninger et al. 2013; Riera et al. 2023). These benefits have been well analyzed as ecosystem services (Lauvie et al. 2023), addressing multiple services such as food and fibers production, pollination, biodiversity conservation, fire prevention, soil fertility, the promotion of local ecological knowledge, and cultural identity, among others (Lamarque et al. 2011; Ruiz-Mirazo and Robles 2012; Oteros-Rozas et al. 2014; Bernués et al. 2014; Rodriguez-Ortega et al. 2014; Hevia et al. 2016).

Agroecology, as a multidimensional approach to land management and agri-food production, seeks to transition from an industrialized system to a sustainable agri-food system that includes both social and environmental sustainability (Wezel et al. 2014; Gliessman 2016). By incorporating innovative livestock practices, from the farm scale to the agri-food system, agroecology seeks to maximize the multifunctionality of pasture-based livestock systems—their ability to deliver diverse ecological and social benefits, beyond the sole production goal (Peeters et al. 2013; Dumont and Bernués 2014). Despite the significant body of research in ecology and environmental disciplines that underscores the role of extensive livestock systems for biodiversity conservation, climate change adaptation, the provision on ecosystem services, little research adopts an agroecological perspective (but see Wezel and Peeters 2014; Soussana et al. 2015; Dumont et al. 2018, 2024). This gap arises from the historical development of agroecology, which has primarily focused on crop production. Traditionally, livestock has predominantly been conceptualized as an additional component to enhance agricultural sustainability, for example, by improving soil fertility through manure integration (Wezel et al. 2014). Consequently, most empirical research on agroecological practices has focused on agriculture (Trichard et al. 2014; De Leijster et al. 2019; Palomo-Campesino et al. 2018, 2022; Boeraeve et al. 2020) rather than livestock farming, despite the urgent need to promote sustainable and multifunctional livestock systems, that provide multiple benefits. Furthermore, studies exploring socio-technical innovations in agroecology and their potential to improve socio-environmental benefits remain scarce (Notenbaert et al. 2021; Ume et al. 2023). To address this research gap, research efforts over the past decade have sought to define agroecological livestock farming as systems designed to produce food and other social–environmental goods while reducing their dependence on external inputs and fostering greater adaptive capacity to face uncertain futures (Dumont et al. 2013; Moulin et al. 2021). Managing livestock systems through an agroecological lens requires designing context-specific practices and strategies that explicitly integrate ecological processes and prioritize social and environmental benefits (Wezel and Peeters 2014; Bonaudo et al. 2014).

In parallel, the socio-environmental benefits of pasture-based livestock systems have been recently recognized as Nature’s Contributions to People (NCP) (Diaz et al. 2018), offering a valuable framework for advancing agroecological transitions in animal production systems. Developed by the Intergovernmental Science-Policy Platform on Biodiversity and Ecosystem Services (IPBES), the NCP framework overcomes the weaknesses identified in the Ecosystem Services Framework by more inclusively integrating the diverse worldviews and knowledge systems that mediate the relationships between humans and nature (Kadykalo et al. 2019). The NCP framework highlights the co-production of nature’s benefits through the integration of natural processes, human activities and anthropogenic capital. NCPs are classified into three categories: material, non-material and regulating contributions. Overall, the NCP framework underlines culture´s pivotal role in mediating human–nature relationships and provides a context-specific perspective for adapting the general NCP categories to each context, giving priority to the integration of local knowledge (Diaz et al. 2018). Thus, it constitutes a robust analytical tool for assessing the benefits of pasture-based livestock systems and pinpointing innovations needed to advancing agroecological transitions (Dean et al. 2021, 2024; Rojido et al. 2024).

Valuation of NCPs provided by agroecological livestock practices can be critical for the sustainable transformation of animal production systems (Dendoncker et al. 2018; Simon-Rojo 2023). However, implementing a single practice, as is often the case with strategies of efficiency or input substitution, generally results in limited changes at both the farm and agri-food system levels. In contrast, adopting multiple practices simultaneously requires a more comprehensive redesign of the system (Wezel et al. 2014). Therefore, recognizing how livestock practices supply NCPs and how they can be bundled based on their functionality could lead to a deeper understanding of the potential of agroecological practices to bring about transformative change and foster agroecological transitions toward sustainable livestock systems.

In this study, we analyze extensive livestock farming experts’ social perception of the NCPs provided by different livestock models and agroecological practices. As a case study, we selected mountainous areas of the Madrid region. Similar to other rural areas in Europe and Spain, they face two major challenges: on the one hand, the expansion of urban sprawl for leisure activities driven by the proximity of the city of Madrid; and on the other hand, the abandonment of farming activities (Plieninger et al. 2006; Gómez-Sal 2011). In recent decades, this context has also facilitated the migration of newcomers from the city of Madrid to the mountains, in some cases, setting up agroecological initiatives with livestock farming at the core (Oteros-Rozas et al. 2017). This phenomenon, known as neo-ruralism and new peasantry, has been reported to be contributing to agroecological transitions (Vizuete et al. 2024).

As a result of the above-mentioned trends, different extensive livestock management models, i.e., livestock systems using the natural resources of the territory, with low use of external inputs and mainly through grazing (Urivelarrea and Linares 2020), coexist in the mountains of the Madrid region. We hereby compare the role of “conventional” and “agroecological” extensive livestock management models (see definitions in the following section), along with the implementation of practices, in the provision of Nature’s Contributions to People (NCPs), to assess their transformative potential in the transition toward sustainable animal production systems in the Madrid region. Our specific aims were: (1) to unravel the differences in the provision of NCPs between the two most prevalent extensive livestock management models in the area; (2) to assess the provision of NCPs by particular livestock practices; (3) to identify similarities and differences among livestock practices in terms of their NCPs provision, and (4) to elicit the potential role of livestock practices in driving agroecological transitions toward sustainable animal production systems.

## Materials and methods

### Study area

The Region of Madrid, located at the center of the Iberian Peninsula, spans an area of 8022 km^2^ and has a population of almost 6.9 million inhabitants, making it the most populated region in Spain (INE 2023). However, the population distribution is highly uneven, with the majority concentrated in the metropolitan area, while many rural villages have fewer than 500 inhabitants (INE 2023). Like the rest of Spain, Madrid’s rural areas have experienced significant changes over the past century, including the intensification of agricultural production and the abandonment of traditional agricultural activities (Camarero 1993; Pinilla et al. 2008).

These trends have also impacted livestock farming, which has historically been practiced extensively in the mountainous areas of northern and western Madrid (the focus of this case study). Since the 1960s, pasture-based livestock farms have sharply declined—by 18% from 1999 to 2009 and by 31% from 2009 to 2020—due to rural depopulation and a shift toward tertiary economic activities such as tourism and recreation (Camarero 1993; INE 2023). This decline has resulted in a predominant livestock model that, although still extensive compared to industrial livestock farming, requires less labor, increases livestock density, diminishes traditional livestock mobility practices and has greater dependence on external inputs, especially animal feed (Ríos-Núñez et al. 2013; Oteros et al. 2017).

Despite this general decline, some neo-rural populations began moving from the city of Madrid to the mountainous areas in the 1990s, taking over the management of some of the remaining small ruminant herds, which included native breeds. These newcomers tend to follow the principles and values of agroecology (Van der Ploeg 2012; Monllor 2013), leading to livestock management practices that differ from those of conventional farmers and generating distinct impacts on the territory (Vizuete et al. 2024, 2025a).

**Fig. 1:**
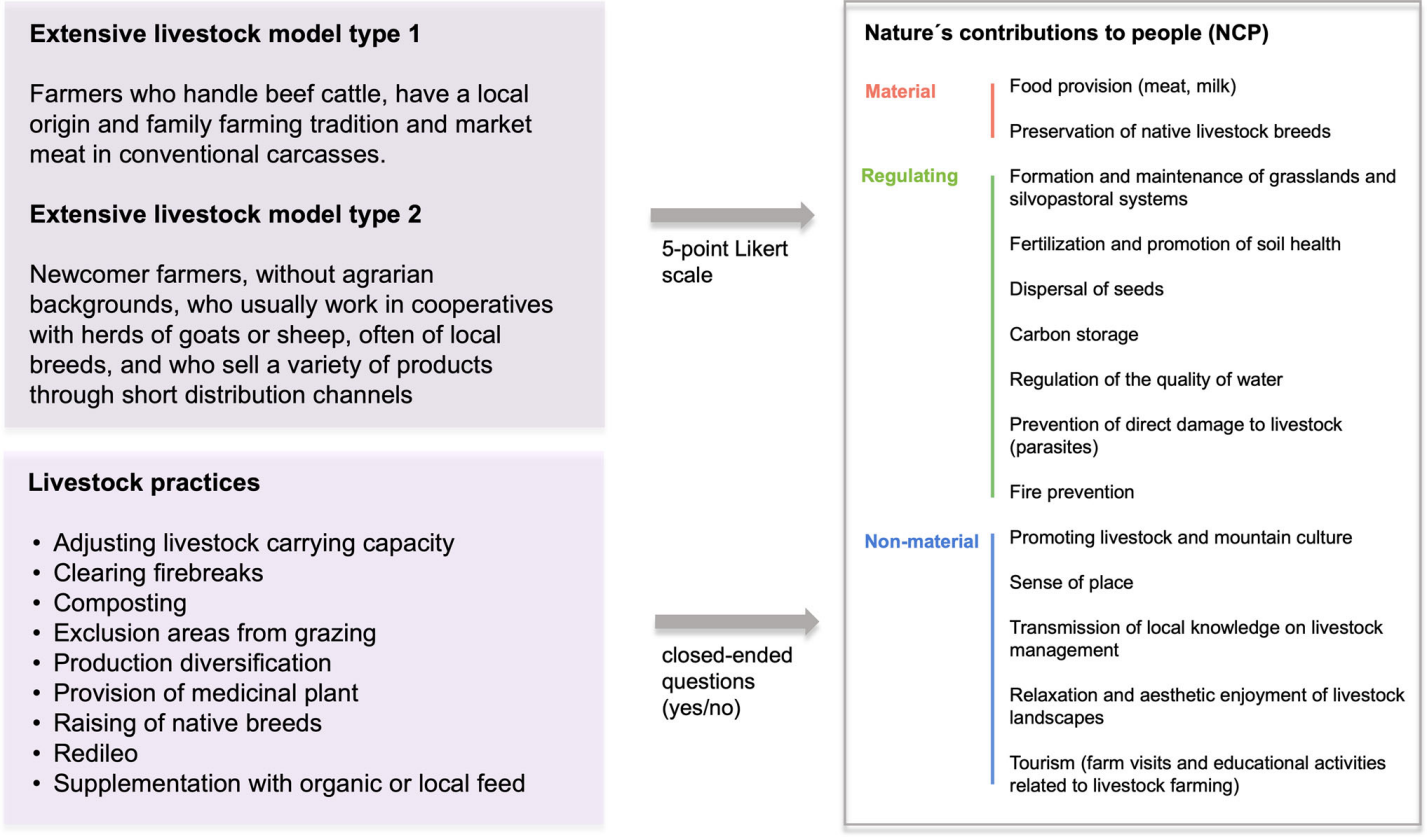
Conceptual scheme of the survey sections

Today, extensive livestock farming in Madrid is concentrated in Sierra de Guadarrama, a mountainous area consisting of cultural landscapes (Plieninger et al. 2012), formed by extensive wood pastures of oak (*Quercus rotundifolia*, *Q. pyrenaica*) and ash (*Fraxinus angustifolia*) trees, patches of local forest and abandoned lands. We selected Madrid as a study area because of the diversity of extensive livestock management models present, along a gradient of management intensity ranging from agroecological to conventional livestock farms (Urivelarrea and Linares 2020). Previous studies on extensive livestock farming in Madrid have shown the existence, in general terms, of two clearly differentiated livestock farming models (Oteros-Rozas et al. 2017). We based our research on these two types of extensive livestock farming models identified in the region by Oteros-Rozas et al. (2017), hereafter referred to as: conventional extensive livestock farming, i.e., the local population that raises cattle and distributes meat through conventional channels, and agroecological extensive livestock farming, i.e., newcomers who raise small ruminants and distribute a variety of products through short channels (Fig. 1). To avoid potential biases associated with the connotations of these terms, we referred to them as type 1 and type 2, respectively, in the survey (Fig. 1; Appendix S2).

### Survey design

We collected data on the social perception of NCPs provision through an online survey administered using Google Forms. We designed a questionnaire that was organized in two sections, the survey was conducted in Spanish, and a translated version is presented here (Figs. 1, S1). In the first section, respondents were introduced to the research and the classification of the two extensive livestock models in the area (namely “types 1 and 2”). In this section, respondents were asked to assess the provision of 14 NCPs by each model in terms of their contribution to the society’s well-being, using a 5-point Likert scale (ranging from 1—strongly disagree to 5—strongly agree). In the second section, we asked the respondents which of the 14 NCPs they considered to be linked to each of 9 agroecological livestock practices.

We adapted and developed a description of each type of extensive livestock model and included it in the survey (Table 1). The 14 context-specific NCPs used in this study were developed by adapting the general categories of the most relevant NCPs identified in the literature on extensive livestock farming in Europe (Dean et al. 2021, 2024) to the specific context of extensive livestock farming in the Madrid region. The resulting final list was presented in the survey using the term ‘benefits ‘ rather than ‘NCPs ‘ for ease of understanding (Supplementary information Table S1). The 9 agroecological livestock practices chosen are considered key to designing sustainable ruminant production systems as proposed by Dumont et al. (2013) and Wezel and Peeters (2014) and fit the context of extensive livestock farming in Madrid (Oteros-Rozas et al. 2017). We referred to them as agroecological practices as they all comply with at least one of the five principles of agroecological livestock farming proposed for Dumont et al. 2013 (animal health, reducing external inputs, reducing pollution, enhancing diversity and resilience, conserving biodiversity), although in the survey we referred to them only as “livestock practices” to avoid bias related to the use of the term agroecology.

**Table 1:** Description of the nine agroecological livestock practices included in the survey

Practices	Description
Adjusting livestock carrying capacity	Regulating livestock numbers to adapt them to the ecological limits of the ecosystem and ensure sustainable use of forage by avoiding overgrazing
Clearing firebreaks	Grazing livestock strategically to reduce vegetation and create barriers to limit the spread of wildfires
Composting	Controlled decomposition of animal manure into fertilizer
Exclusion areas from grazing	Maintain ungrazed grassland areas to promote biodiversity conservation during specific periods, such as the flowering season
Production diversification	Diversification of outputs and functions provided by livestock management, including food products (meat and dairy products), firebreak services and agrotourism or educational activities
Provision of medicinal plant	Supplying medicinal plants to livestock or shepherding livestock in areas where medicinal plants grow strategically
Raising of native breeds	Raising native breeds adapted to the local socio-ecosystem, i.e., the *Guadarrameña* goat or the *Colmenareña* sheep
*Redileo*	Traditional livestock management practice in which animals are confined overnight or for short periods of time to promote grazing and manure deposition in specific areas of the pasture
Supplementation with organic or local feed	Supplementing livestock with extra feed of local or organic origin, in addition to pasture

### Data collection

For the selection of participants, purposive and quota sampling was used, i.e., a non-random technique that aims to study a particular field through a sample of experts selected in proportions relating to different categories (Bryman 2012). We used a multisectoral approach to achieve a diverse sample of experts that included farmers, livestock researchers (ecology, animal production, veterinary sciences), public and private sector technicians such as agricultural advisory services and landscape management and civil society, comprising ecological and conservation-focused associations.

Bringing together the expertise of different stakeholders helps to avoid social perception biases by a particular sector allowing the complexity of pastoral systems to be adequately addressed and integrating the socioecological experiences of the various stakeholders (Díaz et al. 2018; Dean et al. 2024). All the respondents fulfilled two criteria: (1) being specialized in extensive livestock farming, i.e., with a professional or non-professional career linked to extensive livestock farming, and (2) being familiar with the context of livestock farming in Madrid, by living, having researched or worked in the region. To identify potential participants (hereafter referred to as experts for the sake of legibility) who met both criteria we relied on previous research of co-authors in the area from the past three decades (co-authors’ names have been anonymized for peer review) as well as personal contacts of key actors in the livestock sector in Madrid.

After pre-testing, we sent the survey to 105 potential participants in July 2024, attached in an invitation including the objectives of the research, the reason for their selection as a potential participant and the confidential nature of the responses (Supplementary information Appendix S1). The final sample was of 65 responses, consisting of researchers (35.4%), public and private sector technicians (30.8%), farmers (23.1%), social movements (4.6%) and others (6.2%). In addition to their primary categorization, some specialists classified themselves in a second sector as well, 4 as technicians and 9 as social movements.

### Data analysis

First, we calculated the mean values of the NCPs provided by the two types of extensive livestock management models based on the rating of NCPs using the 5-point Likert scale. Then, we used the nonparametric Mann–Whitney U test to explore the differences between the type of extensive livestock farming model and the provision of NCPs according to experts’ criteria. Even considering that each NCP can show different natures simultaneously, we classified each NCPs into one wider material, regulating or non-material IPBES domain, based on the previous literature, to support the discussion of results.

To characterize key agroecological management practices according to their provision of NCPs, we conducted a frequency analysis. To assess similarities or differences between agroecological livestock practices according to their provision of NCPs, Spearman’s correlation coefficients were calculated testing the individual correlation between specific NCPs for each pair of practices.

Finally, we developed a hierarchical cluster analysis (HCA) to identify groups or bundles of agroecological livestock practices with similar provision of NCPs. We used the coefficient of Euclidean distances and Ward’s clustering technique to identify the relationship between practices according to NCPs provision (Ward 1963). This analysis assesses the similarity between practices across the complete NCP profile of each practice and groups them according to similar general profiles. The selection of the number of clusters was based on cluster robustness and knowledge of the nature of practices and the extensive livestock context of Madrid. We calculated the average perceived provision of NCPs for each bundle of practices and used rosewind diagrams to visualize them. All statistical analysis was performed in XLSTAT 2024 software.

## Results

### NCPs provided by two models of extensive livestock management

**Fig. 2:**
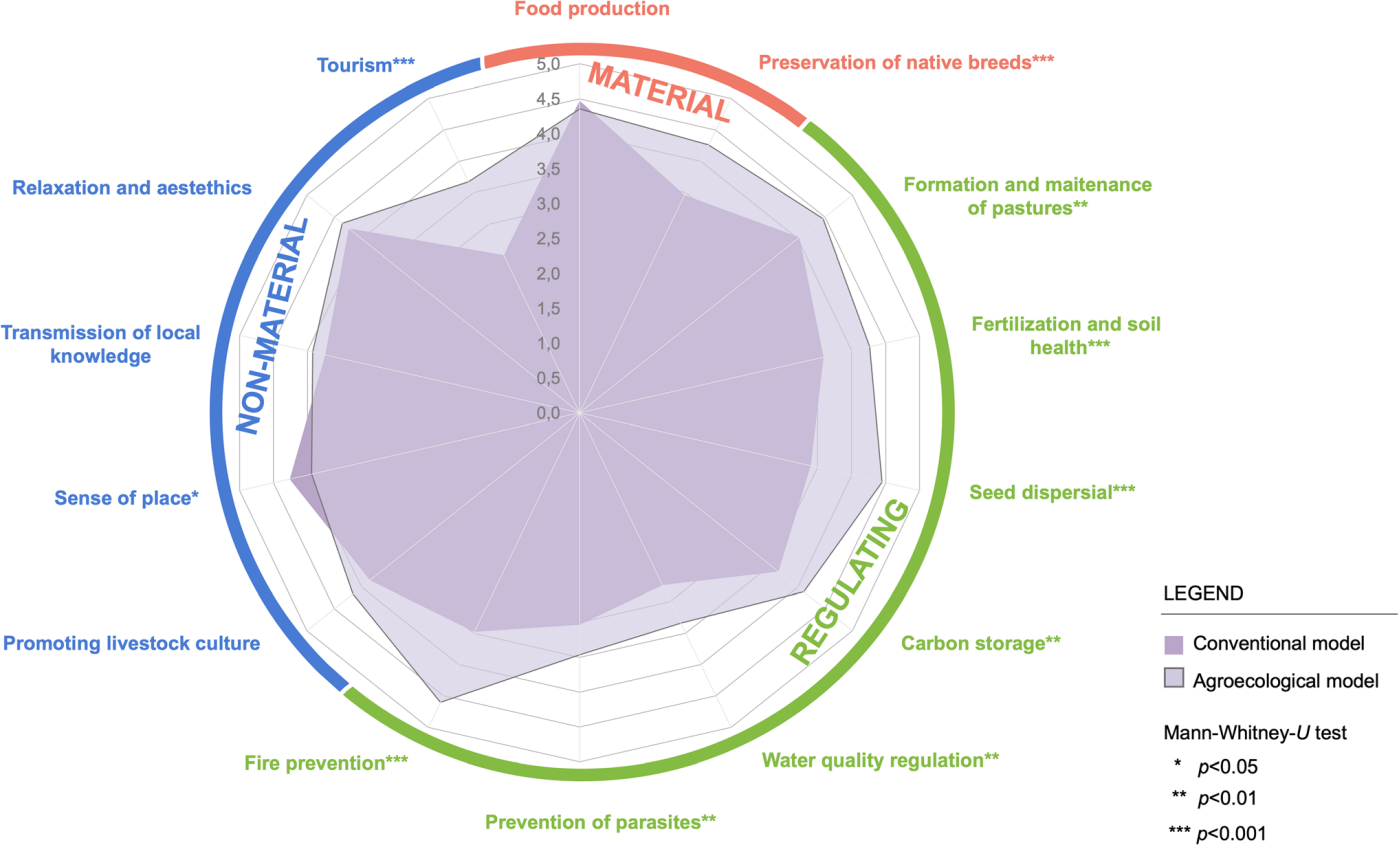
Mean scores of the experts’ perception of the NCPs (material in red, regulating in green and non-material in blue) provided by the conventional (dark purple) and the agroecological (light purple) extensive livestock models, respectively. Significant differences according to the Mann–Whitney U test are marked with *p < 0.05; **p < 0.01; *p* < 0.001

Overall, both types of extensive livestock models, i.e., conventional and agroecological, scored medium and high (all values above 2.5/5) on the provision of all NCPs. Comparing both models, the agroecological model scored higher on all NCPs except food production and sense of place where the conventional model scored slightly higher (Fig. 2; Supplementary information Table S2).

Regarding material NCPs, specialists linked food production a bit higher on the conventional model and the production of native breeds more on the agroecological model. When experts rated regulating NCPs, all scored them higher in the agroecological model. All the non-material NCPs were more related to the agroecological model except sense of place, which was rated higher for the conventional model. Concerning the highest rated NCPs for each model, the extensive livestock conventional model included food production (4.48), sense of place (4.26) and relaxation and aesthetic enjoyment of livestock landscapes (4.23). For the agroecological model, experts assigned the highest scores to formation and maintenance of pastures and silvopastoral systems (4.46), seed dispersal (4.45), food production (4.35), and relaxation and aesthetic enjoyment (4.35).

Although both models scored high, we found statistically significant differences in the social perception of NCPs provided by each extensive livestock management model. The Mann–Whitney test revealed a higher provision by the agroecological model in preservation of native livestock breeds (*U* = 1177; *p* < 0.001), formation and maintenance of grasslands and silvopastoral systems (*U* = 1547; *p* < 0.01), fertilization and promotion of soil health (*U* = 1290.5; *p* < 0.001), dispersion of seeds (*U* = 982.5; *p* < 0.001), carbon storage (*U* = 1497; *p* < 0.01), regulation of the quality of water (*U* = 1427.5; *p* =  < 0.01), prevention of direct damage to livestock (*U* = 1570.5; *p* < 0.01), fire prevention (*U* = 775.5; *p* < 0.001) and tourism (*U* = 856.5; *p* < 0.001). The conventional model provided a higher sense of place (*U* = 2515.5; *p* < 0.05).

### Key livestock practices according to their provision of NCPs

**Fig. 3:**
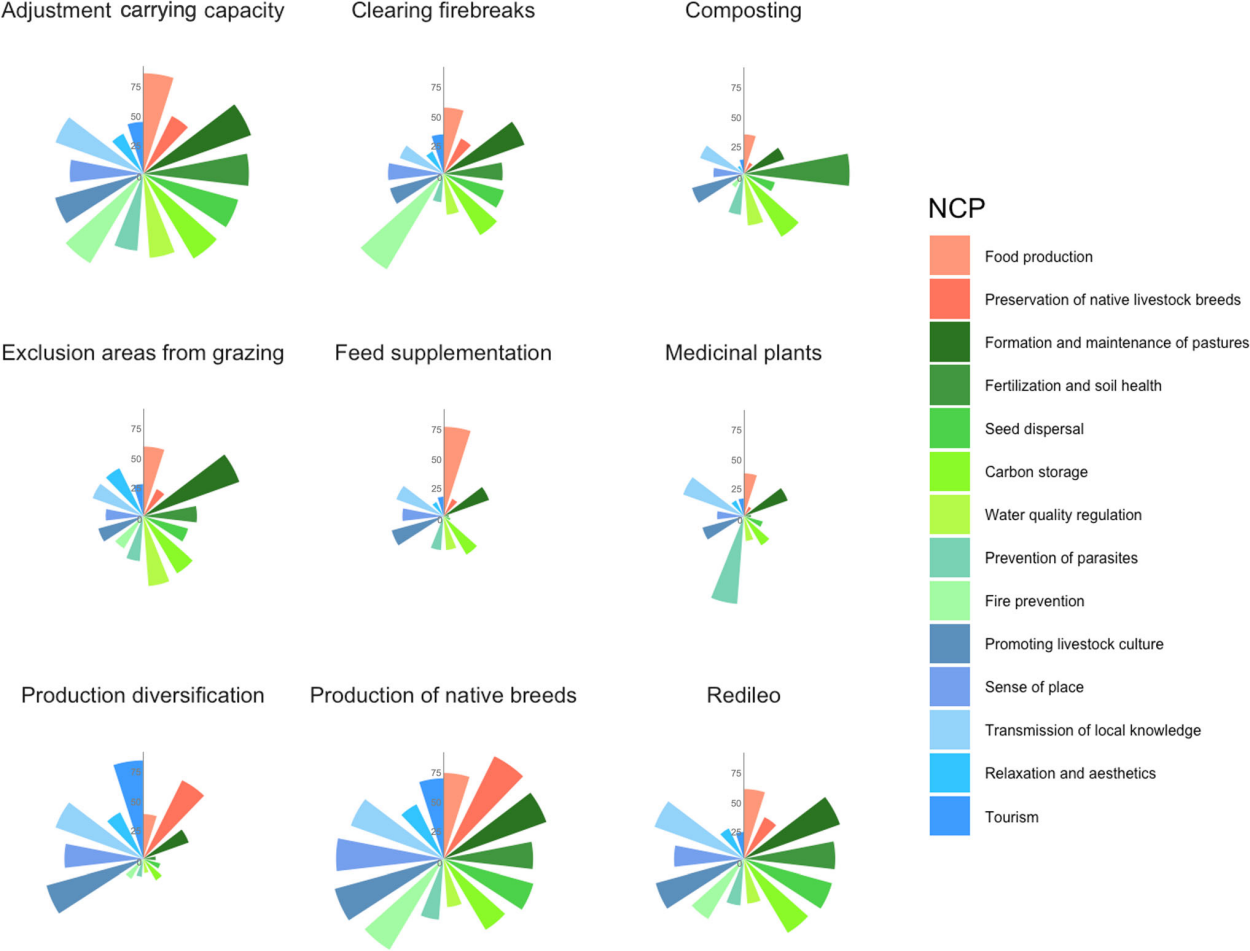
NCPs provided by agroecological livestock practices according to the frequency of affirmative responses from experts. Material NCPs are represented in red tones, regulating NCPs in green tones and non-material NCPs in blue tones. The NCP next to the scale corresponds to food production, and the others are represented according to the order of the legend in clockwise order

Experts associated each practice with the provision of several NCPs. The number of NCPs, as well as types of NCP′s domains provided by the practices, i.e., material, regulating and non-material, varied substantially among the agroecological livestock practices (Fig. 3).

With regard to the number of NCPs per practice, raising native breeds, adjusting livestock carrying capacity and *redileo* (see definitions in Table 1) were the three practices associated with the highest supply of NCPs (11, 11 and 8 NCPs, respectively, by more than 60% of the experts). The three practices with the lowest perceived provision of NCPs were composting, supplementation with organic or local feed and the provision of medicinal plants for livestock (2, 2 and 1 NCP, respectively, by more than 60% of the specialists).

First, regarding material NCPs, both food production and the preservation of native breeds were linked to the practices of native breed production and adjustment of livestock carrying capacity. Additionally, native breed preservation was also linked to production diversification and the production of food with *redileo* and feed supplementation. Second, regulating NCPs were more evenly distributed across practices, except for production diversification, which comprised most of the non-material NCPs. Finally, non-material NCPs were predominantly provided by production diversification, although experts also highlighted the role of *redileo*, the production of native breeds, and adjustment of livestock carrying capacity in supporting identity-related NCPs, i.e., knowledge transmission, livestock culture, and sense of place.

### Similarity in the perception of the associations between NCPs and practices

Out of 36 possible pairs of associations between practices, 9 were positively correlated, meaning they have similar provision of NCPs. We did not find any practice pairs that were significantly negatively correlated (Supplementary information Fig. S1). The highest number of significant positive interactions was found between clearing firebreaks, adjusting livestock carrying capacity and *redileo*, which all shared the provision of regulating NCPs, such as the formation and maintenance of grasslands and fire prevention. The provision of medicinal plants for livestock and supplementation with organic or local feeds showed a strong correlation, as both contributed similarly to non-material NCPs related to livestock culture and knowledge. However, their overall provision of NCPs was lower compared to other practices. The adjustment of the carrying capacity and *redileo* were also correlated due to a high provision of non-material NCPs in addition to regulating NCPs. Composting was a practice related to both *redileo*, exclusion areas from grazing and adjusting livestock carrying capacity, as all received high frequencies in providing carbon storage, and soil fertilization NCPs. Overall, *redileo* and adjusting livestock carrying capacity were positively correlated with more practice pairs than other practices, as they were linked to a greater number and diversity of NCPs, as described above. While no significantly negative correlations were found, the diversification of production exhibited negative relationships with most other practices.

### Bundles of NCPs

**Fig. 4:**
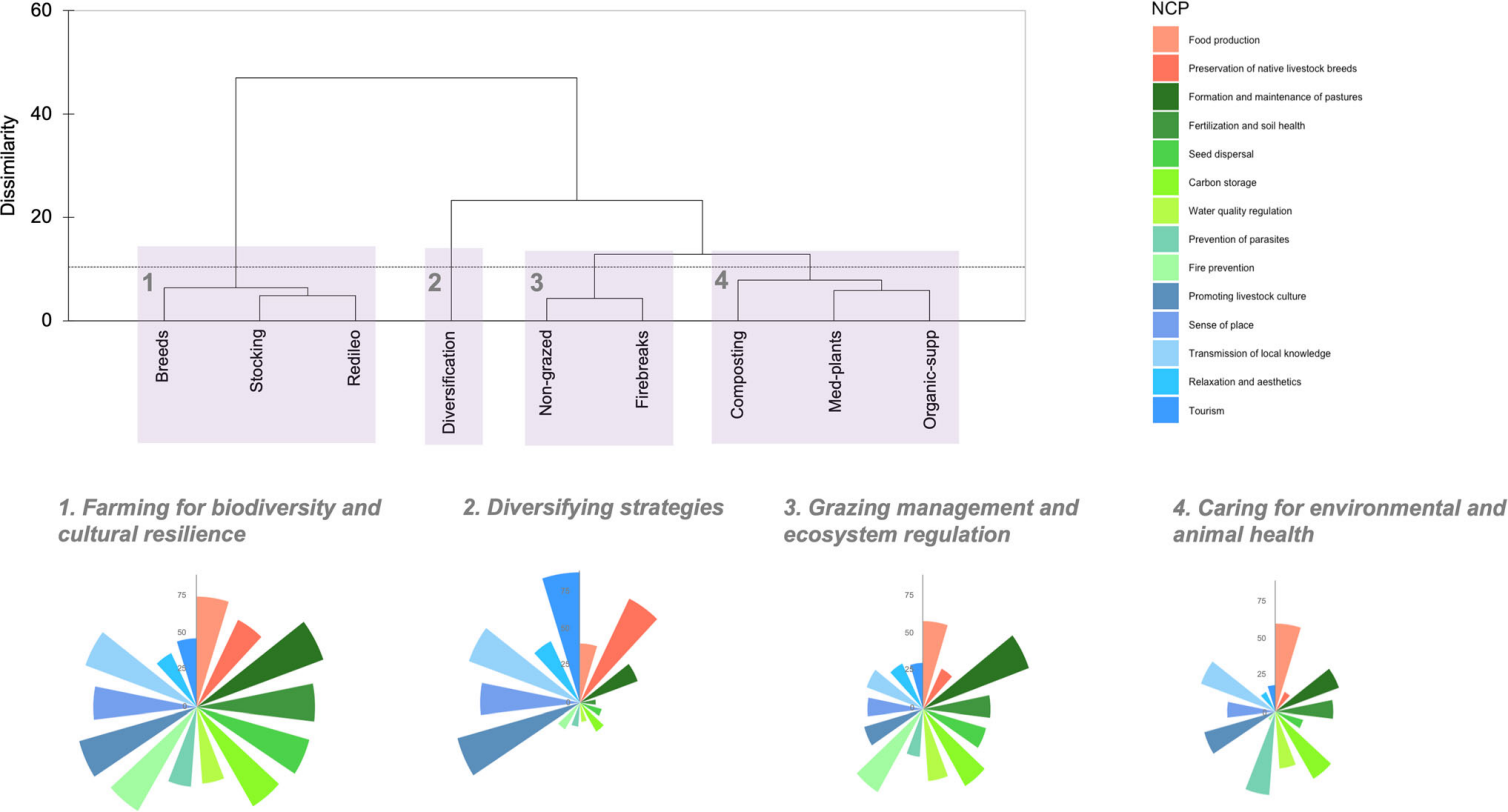
Dendrogram of the hierarchical cluster analysis (HCA). The dendrogram shows bundles of agroecological livestock practices based on their similarities in the supply of NCPs. Material NCPs are represented in red tones, regulating NCPs in green tones and non-material NCPs in blue tones. The NCP next to the scale corresponds to food production, and the others are represented according to the order of the legend in clockwise order

Four bundles of agroecological livestock practices were identified according to their similarity in the perception by experts of NCPs provision (Fig. 4). We named these groups of bundles based on the dominant NCPs and the nature of the practices within each bundle. *Farming for biodiversity and cultural resilience* included animal breeding and landscape management practices through herd movement, such as the production of native breeds, *redileo* and adjusting livestock carrying capacity. All domains of NCPs were high in this group with the regulating NCP of maintenance and conservation of natural habitats and the non-material NCPs of supporting identities with the highest values. *Diversifying strategies* was the smallest bundle, as it only included the practice of production diversification. This bundle stood out in the supply of the material NCP of preserving native breeds and non-material NCPs such as tourism and promoting livestock and mountain culture. *Grazing and ecosystem regulation* included two practices aimed at actively managing grazing, such as clearing firebreaks and exclusion areas from grazing, and excelled in providing regulating NCPs. *Caring for* e*nvironmental and animal health* included practices of composting, provision of medicinal plants and supplementation with organic or local feed.

## Discussion

### Agroecology and extensive livestock farming: A more sustainable model for the provision of NCPs

Agroecology examines the multifunctionality of pasture-based livestock systems to integrate ecological and social benefits, a critical step in sustainable animal production (Peeters et al. 2013; Dumont and Bernués 2014). Applying the NCP framework, this study compares conventional and agroecological extensive livestock models in the Madrid region and evaluates how specific agroecological practices enhance the provision of these contributions. Based on experts’ perceptions, we found a high supply of NCPs from both conventional and agroecological extensive livestock models in the Madrid region, but with significant differences that indicate a higher supply from the agroecological model. Our results show that the agroecological model is associated with greater provision of all regulating NCPs, as well as the material NCP of native breed conservation and the non-material NCP of tourism. These findings are in line with previous research on regenerative farming and new peasantries and confirm the benefits of fostering agroecological initiatives in livestock farming. For example, studies on small ruminants, such as native breeds of sheep and goats, have demonstrated their key role in fire prevention and biodiversity conservation through the maintenance of natural ecosystems, particularly by supporting the recovery of abandoned rural lands and scrub pastures (Serrano-Zulueta et al. 2023; Manzano 2024). In addition, native breeds that are adapted to the territory and graze over large areas or rotate daily between plots, as in the agroecological model, have full access to natural pastures, reducing the need for external feed, which contributes to the regulation of water quality and soil fertilization (Kleppel 2020). Agroecological livestock management also helps prevent parasitic infestations, reducing the veterinary resources needed (Dumont et al. 2013).

Although traditional and local ecological knowledge has been eroded due to the abandonment of pastoral activities and the transition to a market economy (Sharifian et al. 2022), both the conventional and the agroecological model rely on this knowledge system, which explains the shared benefits.

While our results highlight the advantages of agroecological models, certain methodological limitations merit reflection. For example, integrating all NCPs equally as binary variables without units or dimensions of measurement could influence the assessment of multifunctionality), as this approach simplifies complex gradients of provision and may obscure quantitative differences among practices. Nevertheless, previous studies also argue that treating each NCP equally allows for a holistic analysis of how livestock-related NCPs are recognized (Leroy et al. 2024).

### The transformative potential of specific and combined agroecological livestock practices

Our results reveal a variety of correlations between different agroecological livestock practices and uncover four bundles of combined practices. In this section, we discuss how these results contribute to understanding the potential of fostering specific or combined practices to enhance the generation of NCP and to explore the transformative capacity of these practices to guide livestock systems toward greater sustainability.

**Fig. 5:**
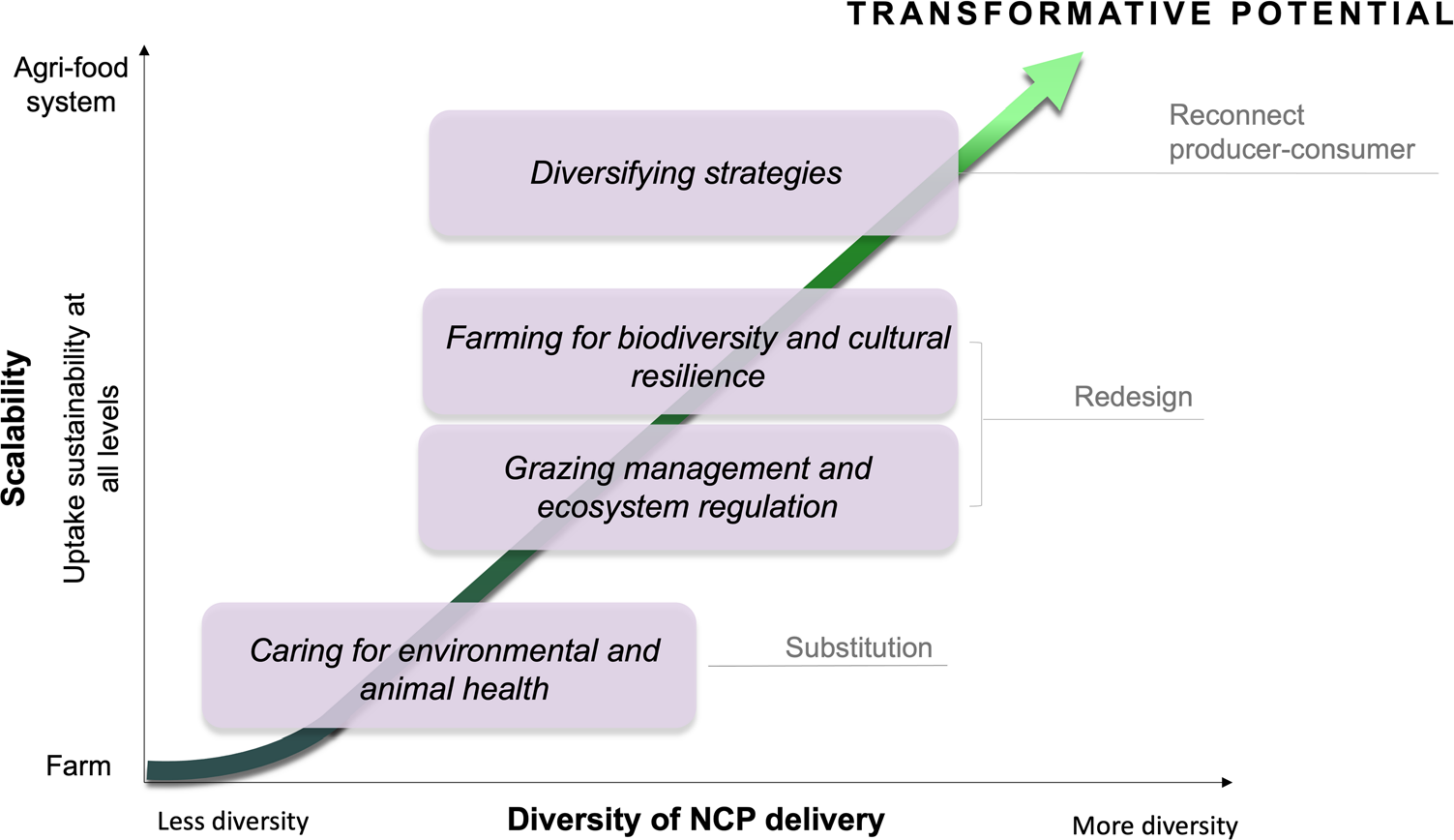
Transformative potential of the agroecological practices identified and bundled according to their NCP delivery and scalability, and their relationship to the increasing levels of agroecological transition proposed by Gliessman (2016), i.e., substitution (farm level), redesign (agroecosystem level) and reconnect producers with consumers (agri-food system level)

Within the *Caring for environmental and animal health* bundle, the combined practices of composting, the provision of medicinal plants and supplementation with organic or local feed do not seem to relate to a wider provision of NCPs by the agroecological model. This is partly because practices such as composting and organic feed supplementation have low adoption rates in the extensive agroecological model due to their economic cost. As a result, many livestock farmers, both conventional and agroecological, choose to produce their own feed and reduce the use of external inputs (Doltra et al. 2018). Similarly, composting and medicinal plant supply have limited adoption in extensive systems. However, the active movement of livestock naturally facilitates spontaneous access to medicinal plants (Brummerloh and Kuca 2023) and overall better livestock health conditions. Additionally, active grazing, common to both conventional and agroecological livestock farming, facilitates distribution of manure and natural fertilization, therefore preventing the need for composting. Therefore, while the transformative capacity of the practices in this bundle is limited within the extensive models studied in this study area, it would be interesting in the future to further disentangle the adoption and transformative potentials of these practices. In fact, replacing conventional inputs with medicinal plants and organic or local feed, along with reducing pollution through manure composting, constitutes an initial step in agroecological transitions (Gliessman 2016; Fig. 5).

The *farming for biodiversity and cultural resilience* bundle, which includes animal breeding and landscape management practices, provided the most diverse pool of NCPs. Our results align with previous studies on the need to recover abandoned traditional ecological practices that foster multifunctionality (Wezel and Peeters 2014), such as *redileo* or the adjustment of stocking rates. Some authors highlight the potential of combining such traditional practices with current socio-technical innovations, a concept called retro-innovations (Stuiver 2006)—as they can validate peasant values and knowledge while integrating innovative alternatives to industrial production models (Van der Ploeg 2012). Indeed, some traditional practices lost in the conventional livestock models are being reintroduced in emerging agroecological systems. A clear example is the management of livestock with virtual fencing, i.e., a non-fixed digital fences implemented by several agroecological initiatives in the study area, such as the *Entrelobas* project^1^. In this project, *redileo*, the adjustment of the stocking rates and the use of local breeds are synergistically integrated with digital fencing through GPS monitoring. As the initiative’s name, “Among female wolves,” suggests, this practice has been linked to the prevention of livestock attacks by wolves, as scrubland grazing opens the landscape and decreases livestock vulnerability to such threat (Pimenta et al. 2018). This synergy between traditional practices and digital tools not only optimizes resource management but also establishes pathways for agroecological transitions aligned with the EU’s Common Agricultural Policy (CAP 2023), which prioritizes resilient, low-impact farming systems enhanced by technological innovations.

Our findings about the *grazing management and ecosystem regulation* bundle highlight the potential of active livestock management practices in the provision of regulating NCPs, such as the maintenance of natural habitats and wildfire prevention. Previous studies have emphasized the importance of moving livestock to maintain the ecosystem structure and biodiversity while preventing overgrazing (Serrano-Zulueta et al. 2023; Manzano 2024). Active management also enables the reconciliation of livestock production with ecological conservation and restoration (Aguilera and Rivera-Ferre 2022). For example, some agroecological practices temporarily exclude livestock from specific grassland areas to promote flowering and pollination, reinforcing ecological dynamics of the ecosystem (Enri et al. 2017). In addition, several regional governments in Spain have implemented policies to promote small ruminant grazing for fire prevention, paying livestock farmers for firebreak clearing services (e.g., Lasanta 2010). Such actions, based on active grazing practices, encourage farm diversification and mobilize values of care for nature and stewardship alongside production activities (Vizuete et al. 2025b).

The *diversifying strategies* bundle groups together practices mostly related to the provision of non-material NCPs. First, in the context of farming homogenization and loss of diversity, product diversification is considered a key practice for increasing farm resilience and reducing dependence on global markets (Tamburi et al. 2020). Second, diversification extends beyond meat and cheese production to also include farm activities such as agro-tourism, educational activities and research collaborations. These activities enhance consumer and local community engagement with farms, fostering trust-based relationships by integrating them as active partners in the different stages of food production (Monllor 2013). Finally, unlike other practices, production diversification enables for a leap in scale from the farm level to the broader agri-food system level, moving beyond input substitution to potential wider agri-food system redesign.

### Toward an agroecological transition of animal production systems

We argue that identifying distinct bundles of practices can help clarify the transformative potential of specific practices. Building on sustainability transformation literature (Gliessman 2016; Lam et al. 2020, 2022; Dabard et al. 2024), we suggest that the ability of agroecological practices to deliver NCPs, as well as the scale at which they take effect (farm or agri-food systems level), might reflect their potential to drive transformative change in agroecological transitions (Fig. 5).

Based on our findings, we hypothesize that livestock farms that incorporate a diverse set of sustainable practices (i.e., that complement each other in the provision of NCPs) both at farm and agri-food system levels may be more prepared for catalyzing transformations under appropriate conditions. *Caring for environmental and animal health* practices, for example, have a limited provision of NCPs and focus on changes at a small level, such as farm or agroecosystem level. Thus, we argue that this may reflect the lower transformation potential of this bundle. The bundles of *farming for biodiversity and cultural resilience* and *grazing management and ecosystem regulation* also integrate practices with the capacity to provide multiple NCPs at small–medium level, i.e., the agroecosystem level, so their potential capacity to generate systemic changes is still limited. Alternatively, the d*iversifying strategies* bundle includes practices, such as agro-tourism or farm-related education activities with the potential to have impact at the agri-food system level and generate multiple NCPs, specifically non-material ones. Broad diversification and the development of alternative food value chains are regarded as fundamental for scaling up and out the agroecology model (Vicente-Vicente et al. 2023), particularly for changes in values and mindsets needed for the preparatory phase of transformative change (i.e., scaling deep, Moore et al. 2015; Pereira et al. 2018; Lam et al. 2022). Yet, the broader transformative impact of diversification strategies depends on their collective adoption and supportive policies and market conditions. Moreover, they may also entail additional workload and management complexity for farmers (Bainville et al. 2025), which should be considered when assessing their scalability and transformative potential.

While our study has highlighted the transformative potential of certain practices for fostering the provision of a more diverse pull of NCPs, we acknowledge that this is a preliminary study with some limitations. First, the sustainable transformation of agri-food systems through agroecology cannot be reduced to a few practices focused solely on agroecosystem redesign (Vicente-Vicente et al. 2023). Building on these insights, future research should explore the valorization of NCPs provided by livestock farming within agri-food systems.

Second, we acknowledge that the study is based on the responses of a limited number of participants, which could be expanded in future research to improve representativeness. Finally, while expert consultation has been applied in various contexts to explore the relationship between agricultural practices and environmental benefits (Dicks et al. 2016; Bernués et al. 2022), it would be interesting to explore other participatory methodologies that capture the complexity of social perceptions about environmental benefits.

## Supplementary Information

Below is the link to the electronic supplementary material.

Supplementary file1 (PDF 352 KB)

## Footnotes

1 https://entrelobas.com/
