# Supplementary material for: Agroecological transition in Madrid’s extensive livestock systems: How expert-perceived NCPs guide transformation pathways
Source: Ambio. 2026 Apr 18;55(9):2266–80. doi: 10.1007/s13280-026-02361-7 (PMC13437822; doi:10.1007/s13280-026-02361-7)
Supplement: Supplementary file 1 [file 13280_2026_2361_MOESM1_ESM.pdf]

Ambio

Supplementary Information

This supplementary information has not been peer reviewed.

Title: **Agroecological transition in Madrid's extensive livestock systems: How expert-perceived NCPs guide transformation pathways**.

Table S1. List of the 14 NCPs selected as the most relevant provided by extensive livestock farming based on previous research in different case studies from around the world (Dean et al. 2021; 2024). These 14 NCPs are context-specific categories adapted from the 18 generalizing categories of NCPs proposed by Diaz et al. (2018).

NCP domain	NCP generalising category	NCP context-specific category	Definition
NCP Regulating			
	1. Habitat creation and maintenance	Formation and maintenance of grasslands and silvopastoral systems	The formation and continuous production of ecological conditions, by ecosystems or organisms within them, necessary or favorable to living organisms of direct or indirect relevance to humans.
	2. Pollination and dispersal of seeds and other propagules	Seed dispersal	Facilitation by animals of the movement of pollen and the dispersal of seeds, larvae or spores of organisms beneficial or harmful to humans.
	4. Regulation of climate	Carbon storage	Climate regulation by ecosystems (positive or negative effects on greenhouse gas emissions).
	7. Regulation of freshwater and coastal water quality	Regulation of freshwater quality	Regulation by ecosystems or specific organisms, of the quality of water through filtration of particulates, pathogens, excess of excess nutrients and other chemicals.
	8. Formation, protection and decontamination of soils and sediments	Fertilization and promotion of soil health	Creation and maintenance of soil structure and fertility by plants, soil organisms and animals.
	9. Regulation of hazards and extreme events	Fire prevention	Ecosystem mitigation of impacts caused by, for example, floods, fires, wind, storms, etc.
	10. Regulation of detrimental organisms and biological processes	Regulation of detrimental species	Regulation, by organisms, of pests, pathogens, predators or competitors that affect humans or plants or animals of importance for humans. Also the direct detrimental effect of organisms on humans or their plants, animals or infrastructure.
NCP Material			
	11. Food and feed	Food provision	Production of food from wild, managed or domesticated organisms.

	14. Medicinal, biochemical and genetic resources	Preservation of native livestock breeds	Production of materials derived from organism and genes and genetic information for plant and animal breeding.
NCP Non-Material	15. Learning and inspiration	Transmission of local knowledge on livestock management	Acquisition of knowledge and development of skills through activities in nature, in contact with ecosystems and organisms, animals and plants.
	16. Physical and psychological experiences	Relaxation and aesthetic enjoyment of livestock landscapes	Provision, through ecosystems or organisms, plants and animals of opportunities for psychologically healing, relaxing activities and aesthetic enjoyment based on close contact with nature.
		Tourism	Provision, through ecosystems or organisms, plants and animals, of opportunities for recreational, leisure and tourism activities.
	17. Supporting identities	Promoting livestock and mountain culture	Activities developed in nature, ecosystems or with organisms, plants and animals as a basis for cultural attachment, traditions, and social identification with nature.
		Sense of place	Opportunities provided by nature for people to develop a sense of place, belonging or home.

Appendix S1. Invitation sent to potential participants, including the objectives of the research, the reason for their selection as potential participants and the confidential nature of the responses.

Survey about the benefits of extensive livestock farming in Madrid

My name is [mane of the first author], I am a researcher at the [name of the university] and a resident of the village of [name of the village] in the Madrid region. In my PhD, together with [name of two of the co-authors] and from the [name of the project] we want to explore the benefits of extensive livestock farming in the Central System of the Madrid region.

We want to know the perception and valuation of people working in the field of extensive livestock farming about the social, economic and environmental benefits associated with different practices and management in extensive livestock farming.

You have been selected to complete the survey because we consider that you have extensive knowledge in this field and that your opinion can be valuable for our research. Participation is voluntary and data will be anonymized so that respondents cannot be identified. The information you provide will be kept confidential. We do not seek to evaluate the knowledge of the participants in any way, as there are no right or wrong answers. What is important is to know your opinion on the questions asked. Please fill in all the questions and complete the survey, otherwise we will not be able to use the data. The estimated time to complete the survey is 10-15 minutes.

If you know any person linked to extensive livestock farming in Madrid and you think their participation could be relevant, please send him/her this link.

If you have any questions, please write to me at [email address of the corresponding author]

Thank you for your time!

Appendix S2. Definition of the two types of extensive livestock farming models identified in the region by Oteros-Rozas et al. (2017) as they appeared in the survey.

Extensive livestock model type 1

Beef cattle farms, feeding mainly on pasture but also with some supplementation. Animals are usually sold to feedlots for the commercialization of the meat, mainly through conventional distribution chains and without distinction (although there are more and more farms with organic certification).

These types of livestock farms are usually managed by people with local origins and middle or old age, who have always lived in these rural areas, have dedicated their whole lives to livestock farming, most of their family income comes from livestock farming (both from production and subsidies) and they usually have a family background in livestock farming. These ranchers care for livestock almost every day of the week but need only a few hours a day.

Extensive livestock model type 2

Herds of sheep or goats, often of native breeds, that feed mainly on grass and, if supplemented, on organic feed or home-grown fodder. The aim of these livestock farms is the production of meat, milk, cheese and yogurt for direct sale, barter and/or self-consumption.

This type of livestock farming is usually managed by young people, who have not always lived in the area (they are often of urban origin) and have no family background in livestock farming. In these livestock farms, associated with a vocation to work on the land and with the animals, the presence of women is also more frequent. This model requires many hours a day of active grazing.

Table S2. Average scores given by specialists to the NCPS provided by each extensive livestock model (SD: Standard Deviation). Significant differences according to the Mann-Whitney-U test are marked with * $p < 0.05$, ** $p < 0.01$ and *** $p < 0.001$.

NCP Domain	NCP	Conventional	Agroecological	Mann-Whitney U
Material NCP	Food provision (meat, milk)	4,477 (0,664)	4,354 (0,759)	2275.5
	Preservation of native livestock breeds	3,462 (1,032)	4,262 (0,796)	1177***
Regulating NCP	Formation and maintenance of grasslands and silvopastoral systems	4,031 (0,935)	4,462 (0,772)	1547**
	Fertilization and promotion of soil health	3,585 (0,983)	4,262 (0,735)	1290.5***
	Dispersal of seeds	3,400 (1,087)	4,446 (0,708)	982.5***
	Carbon storage	3,646 (0,856)	4,108 (0,868)	1497**
	Regulation of the quality of water	2,738 (1,004)	3,338 (1,020)	1427.5**
	Prevention of direct damage to livestock (parasites)	3,031 (0,951)	3,462 (1,017)	1570.5**
	Fire prevention	3.477 (0.954)	4.607 (4.600)	775.50***
Non-material NCP	Promoting livestock and mountain culture	3,846 (1,004)	4,154 (0,870)	1742.5
	Sense of place	4,262 (0,756)	3,938 (0,899)	2515.5*
	Transmission of local knowledge on livestock management	3,738 (1,004)	3,923 (0,941)	1904.5
	Relaxation and aesthetic enjoyment of livestock landscapes (pastures, meadows, stone walls)	4,231 (0,766)	4,354 (0,759)	1907.5
	Tourism (farm visits and educational activities related to livestock farming)	2,508 (1,017)	3,677 (0,920)	856.5***

Figure S1. Correlation matrix of pairs of livestock practices using Spearman's correlation test. Blue and red colors represent positive and negative correlations, respectively. Significant relationships are shown with * $p < 0.05$, ** $p < 0.01$, or *** $p < 0.001$.

	Raising native breeds	Medicinal plants	Supple-mentation	Com-posting	Adjustment stocking capacity	Exclusion areas from grazing	*Redileo*	Clearing firebreaks	Production diversification
Raising native breeds	1	-0,039	0,166	-0,053	0,260	-0,293	0,559*	0,384	0,461
Medicinal plants		1	0,820***	0,388	0,090	0,420	0,363	-0,024	0,185
Suplemmentation			1	0,372	0,084	0,445	0,325	0,104	0,447
Composting				1	0,568*	0,567*	0,588*	0,359	-0,332
Adjustment stocking capacity					1	0,527	0,753**	0,875***	-0,457
Exclusion areas from grazing						1	0,391	0,355	-0,347
Redileo							1	0,644**	-0,026
Clearing firebreaks								1	-0,258
Production diversification									1
